# Supplementary material for: Are carbon emissions trading and green financial instruments synergistic? -Comprehensive quantitative research based on content analysis
Source: PLoS One. 2024 Mar 7;19(3):e0298601. doi: 10.1371/journal.pone.0298601 (PMC10919601; doi:10.1371/journal.pone.0298601)
Supplement: S1 Data — (ZIP) [file pone.0298601.s001.zip › Data/地级市Z_F工作报告.zip!/╝¬┴╓/│ñ┤║╩╨/└┤╫╘╣1⁄2╓┌║┼í╛┬φ┐╦╩2╛▌═°í┐_2017.docx]

各位代表：

　　现在，我代表市政府，向大会报告工作，请予审议，并请市政协各位委员提出意见。

　　一、2016年工作回顾

　　2016年，面对严峻复杂的发展环境，在市委的坚强领导下，我们紧紧依靠和团结全市人民，解放思想、抢抓机遇、创新发展，圆满完成了经济社会发展主要预期目标，实现“十三五”的良好开局。

　　——经济运行稳中向好。预计地区生产总值完成5860亿元，增长7.5%，增速比上年提高1个百分点。地方级财政收入完成415.5亿元，增长7%，增速比上年提高9.3个百分点。规模以上工业产值、固定资产投资、社会消费品零售总额等主要指标增速均比上年有较大幅度的提高。

　　——发展新动能加快成长。改革创新持续推进，战略性新兴产业产值占工业比重提高1个百分点，电子商务交易额增长35%，大众创业、万众创新蓬勃发展，新的增长动力加快形成并不断蓄积力量。

　　——城市品质稳步提升。伊通河百里生态长廊建设扎实推进，旧城改造提升工程启动实施，交通秩序整治成效明显，空气质量优良天数由237天增加到291天，长春荣获“国家森林城市”称号。

　　——区域发展格局不断优化。长春新区建设高点起步、顺利开局，开发区转型升级步伐加快，城区服务业不断进档升级，县域经济加速崛起，新型城镇化取得新成效。

　　——各项事业协调发展。教育、科技、文化、卫生、体育、广播电视等领域取得新进步，安全生产、市场监管、社会治安得到加强，社会大局和谐稳定。

　　——人民生活持续改善。幸福长春行动计划全面完成，为群众办了一批好事、实事。城乡居民增收“暖流计划”启动实施。全年新增就业12.6万人，超出年初预期目标。长春连续九次获评“最具幸福感城市”。

　　一年来，主要做了以下工作：

　　（一）推动产业转型升级，促进经济提速增效。坚持把扩大有效投资摆在经济工作首位，以项目为载体转方式、调结构、补短板，推动经济稳定回升向好。全年实施亿元以上项目1150个、10亿元以上项目284个。完成固定资产投资5000亿元，增长16.7%，增速比上年提高4.7个百分点。启动建设五大现代农业实验区，带动农业发展方式加快转变。综合农机化率超过80%。调减籽粒玉米面积22.3万亩，“长春大米”等优质农产品品牌影响力持续扩大，长春成为首个“创建全国绿色有机农业示范市”。畜牧业稳定发展，免疫无口蹄疫区建设基本完成。制定了工业转型升级实施意见，一汽大众奥迪Q工厂、EA211发动机、长客动车组检修基地、中粮10万吨液糖等一批项目顺利实施，汽车、农产品加工、轨道客车三大支柱产业整体素质不断提升，长春被国家正式列为“中国制造2025”试点示范城市。规模以上工业产值增长8.7%，增速比上年提高20.5个百分点，总量在15个副省级城市中位次由第10位上升到第9位。实施服务业攻坚，出台系列扶持政策，加快建设商业综合体、特色街区、现代服务业集聚区，服务业占GDP比重提高1.6个百分点。长春获批国家服务业综合改革试点。成功举办汽博会、农博会、电影节、雕塑展、雪博会。民航进出港人数增长11%。旅游业总收入增长25%。社会消费品零售总额增长10%，增速比上年提高1.2个百分点。金融业稳步发展，获批筹建东北首家民营银行。互联网和相关服务营业收入增长2 倍。长春进入“中国服务外包示范城市”行列。启动创建国家自主创新示范区、国家级“双创”综合性示范区，加快培育发展新动能。单克隆抗体、玄武岩纤维等一批关键技术实现产业化，航天信息、新能源汽车等10大产业园区启动建设。科技大市场建成开业。全市技术合同交易额达到109亿元，增长3.5倍。3户企业上市，17户科技型中小企业新三板挂牌。国家级众创空间、科技企业孵化器分别发展到9家和49家。国家高新技术企业和科技型“小巨人”企业分别发展到285户和453户。

　　（二）加大改革开放力度，经济发展活力不断增强。扎实推进供给侧结构性改革，努力扩大对外开放，不断破除束缚发展的体制机制障碍，向改革开放要动力、要潜力、要红利。全面启动“一门式、一张网”综合改革，构建“互联网+政务服务”新模式，方便群众和企业办事创业。行政审批中介服务事项削减23%，行政审批要件精简21%。基本建设项目实行并联审批，审批时限压缩到51天。探索推行“双随机、一公开”监管，随机抽取检查对象，随机选派执法检查人员，及时公布查处结果，确保市场活而不乱。认真落实减免税优惠政策，全面推行营改增，进一步清费减负，行政事业性收费和政府性基金实行清单管理，全年减轻企业税费负担30多亿元，经济发展软环境不断改善。全年新增规模以上工业企业273户、规模以上服务业企业793户、限额以上批零住餐企业434户。全社会创业创新热情日益高涨，新登记各类市场主体增长18.3%，其中新登记民营企业户数增长27.1%。民营经济综合配套改革示范区被列为国家试点。淘汰煤炭落后产能117万吨、钢铁落后产能48万吨。汽车库存降低2.6万辆。商品住房可售面积下降15%。市属国有企业股权规范重组取得积极进展，开展公共资源交易平台整合，供销社、农村金融、不动产登记、集体经营性建设用地入市等领域改革有序推进。抢抓国家“一带一路”、新一轮东北老工业基地振兴等重大机遇，主动参与中蒙经济走廊建设，积极与天津市开展对口合作，兴隆综保区获批国家一类口岸，“长满欧”货运班列实现常态化运行。中德、中韩、中白等国际合作产业园区加快建设，华侨华人创新创业聚集区落户长春。引进了华为云计算、浪潮大数据等一批高质量的大项目，实际利用内外资分别增长15%和12%。

　　（三）提高建设管理水平，打造绿色宜居新型城市。坚持新区建设与旧城改造并重，强化城市规划、建设、管理，推动城市承载能力、形象面貌稳步提升。以城市发展战略规划为统领的30个专项规划实现“多规合一”，基本实现主城区“规划一张图”。伊通河百里生态长廊建设扎实推进，城区段主河道完成截污，南溪湿地、沿河旅游设施、防洪生态绿道、调蓄池、污水处理厂建设顺利推进，建成区黑臭水体治理全面启动。实施三环以内旧城改造提升工程，综合整治6个商圈、155个老旧小区、2087栋楼体，集中打造260条示范街路、规范街路，亮化美化一批重点街路、公园广场。拆除违法建筑1.8万处、193万平方米。完成16个城市出入口综合改造。亚泰大街南四环路立交桥、机场大道哈尔滨大街至保税区段、吉林大路东延长线等一批重要路桥建成通车。新建7座人行天桥，打通32条断头路、卡脖路，打开253处小区围墙。开展整治道路交通秩序大会战，完善道路标牌标线，清理私设地桩、地锁、隔离墩18万个，施划公共停车泊位34万个。地铁1号线热滑试车，地铁2号线、北湖快轨、轻轨3号线延伸线建设进展顺利。建成17.6公里地下综合管廊廊体，改造地下管网822.8公里，外环燃气高压管网全线竣工，哈沈天然气干线连接工程交付使用，城区3个热电厂实现热网联接。加大空气污染防治力度，关停搬迁重污染企业12户，淘汰4万辆黄标车和老旧车辆、1255台燃煤小锅炉，秸秆综合利用率达到75%。提升城市保洁标准，地面清洗范围扩大到26个广场、596条道路，16条街路实施深度保洁。新建6个社区公园、15宗大块绿地。长春世界雕塑公园被国家评定为5A级景区。“长春德苑”主题公园建成开放，文明城创建取得新成效。

　　（四）全力改善民生，不断提升群众幸福感满意度。坚持把民生作为发展的“指南针”，破解民生难题、回应民生关切、满足民生需求，推动民生状况持续改善。强力推进脱贫攻坚，实施各类扶贫项目626个，76.2%贫困人口、74%贫困村脱贫。加大就业创业支持力度，对少裁员、不裁员企业发放2.1亿元稳岗补贴，城镇登记失业率保持在4%以下。提高居民基础养老金、城乡低保、重点优抚对象补助标准，企业退休人员养老保险金实现“十二连增”。全面实施困难残疾人生活补贴、重度残疾人护理补贴制度。建立政府购买居家养老服务新模式，10万名失独、失能、空巢老人受益。拆除90万平方米棚户区，当年安置被拆迁居民1.1万户。改造“暖房子”254.7万平方米。1.7万户群众享受货币和实物住房保障。2.26万户中低收入家庭获得公积金低息贷款。无籍房确权取得新进展。完成7.8万户居民二次供水接收改造。改造农村室内水冲式厕所1.45万户。新建农村公路928公里。23个村获评省级“美丽乡村”。新建5所义务教育学校、22所公办幼儿园，公办小学100%、公办初中90%以上实现划片入学，基础教育质量和均衡化水平稳步提升。市属公立医院取消药品加成，住院费中的药费占比下降15.2个百分点。积极推动分级诊疗，区级医院门诊量增加2倍。在全国率先启动失能人员长期护理保险制度试点，医保低自付、慢性病门诊治疗病种进一步增加，长春与部分省市实现医保即时结算。实施城区残疾人免费乘坐公交车政策。开辟优化公交线路22条，新建港湾式停靠站20座。2000余台公共自行车投入使用。举办市民艺术节、送戏下基层、公益电影放映等活动，公共文化服务均等化水平不断提升。长春体育中心完成改造，启动建设伊通河、南湖等一批健身步道。深入开展幸福社区创建，城市千米社区比例达到67%，农村社区用房面积平均超过400平方米。社区建设经验在全国推广。

　　（五）加强民主法制建设，维护社会和谐稳定。坚持运用民主法治思维推动发展、化解矛盾、维护稳定，不断加强政府自身建设、提升社会治理能力。自觉接受人大及其常委会的法律监督、工作监督和政协的民主监督，坚持重大事项向人大报告、与政协协商制度。认真听取各民主党派、工商联以及无党派人士的意见建议。办理人大议案2件、人大代表建议174件、政协建议案3件、政协提案326件。深入推进依法行政，严格执行重大行政决策法定程序，不断加大政务公开力度，努力保障公众的知情权、参与权。公布市直部门责任清单，实施教育规范、限期整改、依法处罚的“三段式”执法模式，认真落实规范性文件备案审查、重大行政处罚报备制度。加强安全生产监管，安全事故总量、死亡人数分别下降16.8%和15.8%，安全生产形势总体稳定。创建“国家食品安全示范城市”，不断强化食品药品安全监管。加强立体化社会治安防控体系建设，严厉打击各类违法犯罪，110刑事警情下降16.2%，“两抢”案件下降13.2%，刑事案件下降27.5%。人防基础设施不断完善。全面提高局长接待日、读报读网工作水平，市长公开电话在全国地级以上城市服务热线质量测评中获得第二名。强化信访接待，初信初访办结率达到93.6%。启动实施“七五”普法，深入开展法治宣传教育。民族团结、宗教和谐良好局面进一步巩固。军政军民团结奋进，双拥模范城实现“八连冠”。扎实开展“两学一做”学习教育，深入开展以“抢抓机遇、创新发展”为主题的解放思想大讨论，实施电视问政活动，全面强化政务督查，敢于担当、竞进有为的发展氛围更加浓厚。锲而不舍落实中央八项规定精神，坚决纠正“四风”。加强行政监察和审计监督。大力推进党风廉政建设，始终保持惩治腐败的高压态势。

　　根据中央部署，本届政府提前一年换届。市十四届人大一次会议以来，在市委的坚强领导下，我们紧紧依靠和团结全市人民，坚持稳中求进工作总基调，实施一系列利当前、惠长远的重大举措，推动全市经济社会发展不断取得新的成就。

　　这四年，是长春经济适应把握引领新常态、不断改革创新奋力前行的四年。经济运行克服下行压力逐步止跌回稳，重新回到合理区间，在东北率先实现回升向好，城市综合经济实力显著提升。传统制造业提质增效，现代服务业迅速成长，新产业、新业态、新商业模式不断涌现，经济结构加快调整，转型升级迈出坚实步伐。

　　这四年，是城市规模扩张和城市品质提升良性互动、相互促进的四年。新的城市次中心在绕城高速公路以外竞相成长，主城区620多万平方米棚户区、3400多万平方米老旧楼宇经过改造旧貌换新颜。支撑城市发展的立体交通体系框架基本形成，水、电、气、热等生产要素供应保障能力稳步提高，体现空气质量的PM10、PM2.5两项指标连续三年下降。

　　这四年，是保障民生力度持续加大、民生状况稳步改善提升的四年。城乡居民收入增长快于经济增速。城乡养老、医疗保险覆盖率均达到95%以上，初步实现人人享有基本社会保障。低收入群众住房条件、残疾群众康复救助力度、困难群众基本生活保障水平每年都有新提高。经过四年努力，我市经济社会发展迈上了新的台阶。

　　各位代表，长春经济社会发展取得的成绩，是省委、省政府和市委正确领导的结果，是人大、政协以及社会各界大力支持的结果，是全市广大干部群众团结奋斗的结果。在此，我代表市政府，向全市广大干部群众致以崇高的敬意！向人大代表、政协委员和社会各界人士，向中省直驻长单位，人民解放军和武警驻长部队以及海内外的朋友们，表示衷心的感谢！

　　肯定成绩的同时也要清醒看到，长春振兴发展还面临着许多困难和挑战。经济持续稳定向好的基础还不牢固，结构调整转型升级任务繁重，科技创新带动作用尚未得到充分释放，新动能支撑发展的能力仍然较弱，经济发展软环境尚需进一步改善，城市基础设施建设与环境治理任重道远，影响城市安全的潜在风险和隐患依然较多，一些群众关心的热点难点问题还没有得到有效解决。政府职能转变还不到位，管理和服务水平有待提高，廉政建设和作风建设仍需加强。我们要进一步增强忧患意识和担当意识，下更大力气解决这些问题，尽心竭力做好政府工作，不辜负人民期望和重托。

　　二、今后五年主要任务

　　市十三次党代会确立了建设东北亚区域性中心城市的宏伟蓝图。我们要立足新起点、瞄准新目标、实现新跨越，谱写长春振兴发展的新篇章。

　　今后五年政府工作的指导思想是：以马克思列宁主义、毛泽东思想、邓小平理论、“三个代表”重要思想、科学发展观为指导，全面贯彻党的十八大和十八届三中、四中、五中、六中全会精神，坚持以习近平总书记系列重要讲话精神和治国理政新理念新思想新战略统揽经济社会发展全局，认真践行“五位一体”总体布局、“四个全面”战略布局和“五大发展”理念，坚持“稳中求进”工作总基调，全面落实省委“三个五”发展战略和“打先锋、站排头”要求，按照市十三次党代会的战略部署，以全面振兴为主线，突出吉林中部创新转型核心区建设，努力在率先全面建成小康社会进程中，加快建设东北亚区域性中心城市。

　　今后五年，我们要打造经济量级、城市能级、民生改善、社会治理和生态文明五个“升级版”，努力做到“五个一”：“一个高于”,主要经济指标增速持续高于全省平均水平；“一个前移”，城市综合竞争力在全国同类城市中实现位次前移；“一个领先”，生态文明建设保持全国领先水平；“一个率先”，在全省率先全面建成小康社会；“一个提升”，党建科学化水平全面提升。努力实现六个目标：

　　——经济发达。毫不动摇坚持以经济建设为中心，努力保持经济中高速增长，推动产业迈上中高端水平，不断提升城市经济综合实力、核心竞争力。经济规模加快向“万亿俱乐部”迈进，地方级财政收入突破600亿元。三次产业结构持续优化，由“二三一”向“三二一”转变。全力建设世界级汽车、农产品加工、轨道客车研发制造服务基地，努力打造先进装备制造、生物及医药健康、光电信息、新能源汽车、新材料、大数据6个千亿级产业。国家重点实验室达到20个，国家企业技术中心达到8个，国家高新技术企业力争达到1000户，科技型“小巨人”企业新增1000户，全社会研发经费投入强度达到3%以上，成为国家创新型城市。人均GDP超过1.5万美元，长春进入中等发达城市行列。

　　——文化繁荣。实施“文化兴市”战略，努力推动社会主义文化大发展大繁荣。“中国梦”和社会主义核心价值观深入人心，爱国主义、集体主义、社会主义思想广泛弘扬，市民思想道德素质、科学文化素质、健康素质明显提高。文化与旅游、科技、金融、互联网深度融合，文化产业占GDP比重达到5%以上，成为新的支柱产业。基本建成覆盖城乡、便捷高效、保基本、促公平的现代公共文化服务体系。文化软实力、影响力全面增强，城市知名度、美誉度明显提升，长春进入国家历史文化名城行列，实现全国文明城市“三连冠”。

　　——法治优良。全面推动依法治市，努力提高城市治理体系和治理能力现代化水平。政府活动全面纳入法治轨道，切实做到法定职责必须为、法无授权不可为，自觉运用法治思维和法治方式推动工作。平安建设扎实推进，治安防控体系日益完善，群众安全感不断提升。社会矛盾纠纷化解机制进一步健全，法律援助能力不断增强，群众合法权益得到有效维护。市场主体依法经营，企业社会责任普遍增强，城市信用环境持续改善。群众法律素养、法治意识明显提高，尊法守法成为全体市民的共同追求和自觉行为。社会在深刻变革中既生机勃勃又井然有序，长春成为公平公正安定有序的一流法治城市。

　　——功能完善。坚持以强烈的文化意识引领规划、建设、管理，推动城市发展方式由外延式扩张向内涵式发展转变。城乡一体化进程不断加快，常住人口达到800万左右，城镇化率达到68%左右，中心城区规划建成区面积达到450平方公里。快速路体系、轨道交通网络、慢行系统全面形成，畅通水平稳步提升。老旧地下管网基本完成改造，水、电、气、热等生产要素能够满足当期需求并适度超前。基本完成棚户区改造，加大老旧小区、历史文化街区改造力度，城市形象全面提升。信息技术在城市管理中得到广泛应用，新型智慧城市建设取得实质性进展。城市辐射、集散、带动能力不断扩大，成为区域性生产要素配置中心、信息资源交流中心和高端商务活动聚集中心。

　　——生态一流。创新环境治理理念和方式，实行最严格的环境保护制度，加快形成绿色生产生活方式，不断提升资源利用效率和生态环境质量。生态建设约束性指标全面达标，主要污染物排放总量显著下降，空气质量优良天数比率超过80%。城区绿地覆盖率达到42%以上，全市森林覆盖率稳步提升。符合海绵城市标准的建成区面积达到20%。伊通河城区段成为百里生态长廊。市域河流断面水质达到国家考核标准。全民节约意识、环保意识、生态意识明显增强，循环经济、低碳经济等绿色经济模式基本形成，天蓝、地绿、水净、城美、空气清新成为常态。

　　——人民幸福。全面落实以人民为中心的发展思想，着力解决群众最关心最直接最现实的利益问题。教育、体育、医疗、住房等公共服务体系更加健全，基本公共服务均等化水平稳步提高。就业比较充分，城镇登记失业率持续低于全国平均水平。城乡居民收入与GDP同步增长，收入差距不断缩小，中等收入人口比重稳步提高。劳动年龄人口平均受教育年限提高0.5年，人均预期寿命提高1岁。社会保障制度更加公平、更可持续，养老、医疗保险覆盖面达到97%以上。社会救助体系更加完善，困难群众得到更有针对性的帮扶。农村贫困人口、贫困村全部脱贫。全市人民群众获得感、满意度全面提升，共同迈入全面小康社会。

　　三、2017年重点工作

　　今年是新一届政府开局之年，我们要把长春发展业已形成的良好势头巩固和持续下去，争取更大的发展、更多的成绩。全市经济社会发展的主要预期目标是：地区生产总值增长7.8%左右，规模以上工业产值增长8.3%以上，全社会固定资产投资增长15%以上，社会消费品零售总额增长10%以上，地方级财政收入、城乡居民收入与经济保持同步增长，居民消费价格涨幅3%左右，城镇登记失业率控制在4%以内。在实际工作中，我们要以奋发有为的状态、争先进位的勇气、攻坚克难的决心，努力争取更好的结果。

　　做好今年经济社会发展工作，必须全面贯彻新一轮东北振兴战略。去年以来，党中央、国务院围绕东北振兴连续下发三个文件，操作性强、含金量高。要紧密结合实际，全面对接、精准发力、狠抓落实，把每一条政策措施用好、用足、用到位。必须牢牢把握稳中求进工作总基调。稳是大局，进是目的。在确保经济增长稳定、社会大局稳定、市场预期稳定的前提下，力争在结构调整、改革开放、城市建设、生态环境、改善民生等关键领域奋发有为、有所进取，一步一个脚印向前迈进。必须以供给侧结构性改革作为主线。供给侧结构性改革刚刚开始，更艰巨的任务还在后面。要统筹推进“三去一降一补”，既去旧、又育新，既减少无效供给、又扩大有效供给，努力推动经济朝着更高质量、更有效率、更加公平、更可持续的方向发展。重点做好九个方面工作：

　　（一）促进经济持续稳定增长。要采取有力措施，挖掘增长潜力、激发市场动力、释放企业活力，推动经济稳定持续向好。充分发挥投资的关键作用，加大对基础设施、科技创新、生态环保等领域政府投资，引导和撬动社会资本更多投向制造业、战略性新兴产业和现代服务业。加强项目谋划储备，加快项目前期工作，落实项目建设条件，继续推行重大项目服务秘书制和领导包保责任制，全年实施亿元以上项目1200个。深化投融资体制改革，推动融资平台转型进行市场化融资，探索基础设施等资产证券化。规范政府举债行为。支持企业上市、新三板挂牌。切实发挥消费的基础作用，支持发展养老、健康、家政、教育培训、文化体育等服务消费，壮大网络信息、智能家居、个性时尚等新兴消费。深入实施消费品“增品种、提品质、创品牌”专项行动，推动消费升级。支持企业债转股，加大股权融资力度，切实降低企业杠杆率。在落实税收优惠政策、清理涉企收费、降低要素成本上下功夫，规范认证、评估、检查、检测等中介评估收费，切实减轻企业负担。深入推进“放管服”改革，全面推广“双随机、一公开”监管模式，全面优化企业发展软环境。强化重点企业跟踪包保措施，加强生产要素组织调度。加大对中小企业扶持力度，有针对性地解决实际困难。规模以上工业、服务业企业分别新增150户、500户以上。

　　（二）努力培育发展新动能。把创新摆在发展全局的核心位置，深入创建国家自主创新示范区和国家级“双创”综合性示范区，推动新技术、新产业、新业态加快成长。全面落实《大力推进科技创新的实施意见》，支持高校院所重大科技成果就地转化，推动检验检测等科技公共服务平台建设，鼓励企业建立研发中心、并购国内外研发机构和先进科技型企业，政府科技投入增加50%以上。实施10个重大科技攻关、10个重大科技成果转化项目，推动工业机器人、新能源汽车、卫星遥感数据应用产业化不断实现新突破。充分发挥科技大市场、科技金融创新中心作用，把创新成果的研发、中试、转化、推广、应用等环节有效衔接起来，逐步建立科技成果转化的“舟桥”机制。全年新增国家高新技术企业、科技型“小巨人”企业户数力争比上年翻一番。加快建设中德、中白、中韩、中以、中古等中外合作科技园区，续建长东北科技创新中心、北湖科技园。打造一批众创、众包、众扶、众筹等“双创、四众”支撑平台，新增创业孵化面积50万平方米。突出发展民营经济，抓好民营经济综合配套改革示范区国家试点，完善融资担保体系，新登记民营中小企业2万户以上，民营经济主营业务收入增长10%左右。建设人力资源产业园。实施更积极、更开放、更有效的人才引进政策，加快建设“人才强市”。引导企业发扬“工匠精神”，强化质量管理，加强品牌建设，增强产品竞争力。弘扬企业家精神，支持企业家专心创新创业。大力弘扬创新文化，厚植创新沃土，调动全社会创业创新积极性，汇聚成推动发展的磅礴力量。

　　（三）加快产业转型升级。狠抓增量投入，加快调整结构，放手发展规模，努力提高质量，大力振兴实体经济。加快实施“中国制造2025”试点示范城市建设，注重运用新技术新业态改造提升传统产业，提高智能制造、绿色制造、精益制造和服务型制造能力，工业投资增长14%以上。培育红旗乘用车、高寒宽轨高速动车组、聚乳酸等一批新产品，带动三大支柱产业加速向高端化迈进。不断壮大先进装备制造、生物及医药健康、光电信息、新能源汽车、新材料、大数据产业，完成投资增长15%以上。实施服务业发展攻坚，加快建设商业综合体、五星级酒店、综合性物流园区，发展壮大电子商务、服务外包、文化创意、动漫设计等现代服务业集聚区，服务业投资增长16%以上。开展服务业综合改革试点，提高生产性服务业专业化、生活性服务业精细化水平。整合发挥优势资源，不断强化金融、旅游、会展、商贸、教育、医疗、文化区域性服务能力。推进商品房特别是商业楼宇去库存。综合运用金融、土地、投资等手段，促进房地产市场平稳健康发展。积极发展装配式建筑产业。以促进农民增收为核心，深入推进农业供给侧结构性改革。加快建设五大现代农业实验区，促进农业提质增效。把增加绿色优质农产品供给放在突出位置，加强农产品标准化生产、品牌创建、质量安全监管，积极争取创建国家农业可持续发展试验示范区。引导农民瞄准市场调整种植结构。突出发展现代畜牧业。支持农产品精深加工，延伸产业链条，提高产品附加值。强化农业水利基础设施建设。发展适度规模经营，稳步提高土地流转比例、综合农机化率。推动电子商务进农村。

　　(四)深入推进改革开放。深化供给侧结构性改革，围绕解决重点领域的突出矛盾和问题，加快破除体制机制障碍，进一步激发市场活力和社会创造力。向纵深推进“一门式、一张网”政务服务综合改革，实现行政审批和公共服务一号申请、一窗受理、一网通办，革除陈规旧制、砍掉繁文缛节，为群众和企业办事创业开路清障。深化国企国资改革，开展混合所有制改革试点，稳步推进经营性国有资产集中统一监管，推进驻长央企“三供一业”分离移交。推动厂办大集体改革取得实质性进展。加快建设农村金融综合改革试验区，新组建一批村镇银行。继续推进商事制度、科技、教育、文化、医药卫生、地方国有林场以及农村产权制度等领域改革，为发展加力、让群众受益。主动融入国家“一带一路”、“长吉图”、“哈长城市群”，推动新一轮高水平对外开放。全面加大招商引资力度，突出引进国际战略投资者、央企、民营500强企业，实际利用内外资分别增长15%和12%以上。抓好兴隆综保区、空港开发区等对外合作平台建设，大力发展保税物流、跨境电子商务产业，积极争取设立汽车整车进口口岸。推动外贸创新发展，进出口总额增长5%以上。扩大国际产能合作，推动装备、技术、服务走出去。积极与天津市以及浙江省相关城市开展对口合作，通过市场化方式吸引企业来长投资，共建产业合作园区。主动对接京津冀协同发展战略，全面加强与环渤海、长三角地区合作。

　　（五）优化区域发展格局。突出特色、分类指导、统筹兼顾，促进城区、开发区、县域优势互补、错位发展、协同并进。城区要树立集约发展、内涵提升、精明增长理念，加快发展双创空间、特色街区、现代服务业集聚区和都市型工业、高端制造业园区。支持双阳、九台加快发展，尽快成为现代化新城区。长春新区要在体制机制创新等方面先行先试，全面加大招商引资、项目建设、基础设施建设力度，不断提升要素集聚能力，尽快成为带动全市发展的新引擎。经开、汽车开发区要集中精力发展新型工业化，强化创新驱动，壮大特色园区，培育产业集群，成为工业转型升级的主力军。净月开发区、莲花山度假区要集聚创新创业资源，突出发展现代服务业，不断优化生态环境。县域要以更大的力度推进工业化、城镇化，培育有特色的产业集群，按照中等城市标准抓好县城建设，提高教育、医疗等公共服务水平，增强对农业转移人口的吸引力。扎实推进以人为核心的新型城镇化，促进农民工市民化。综合治理农村环境突出问题，建设美丽宜居乡村。新建改造主城区农村公路500公里。全面完成74个贫困村安全饮水工程。建设长春一小时经济圈环线，逐步畅通与伊通、公主岭交通联系，持续推进长吉一体化，强化中心城市集聚扩散能力。落实最严格的耕地保护制度，提升土地节约集约利用水平。

　　（六）提高城市宜居水平。牢固树立人民城市为人民的思想，坚持以人为本、建管并重、生态优先，不断提高城市承载能力、优化城市环境质量。突出规划引领作用，推行空间规划“一张图”管理模式。全面开展城市设计，统筹建筑布局，协调景观风貌，培育城市特色。续建伊通河百里生态长廊，基本完成南溪湿地建设、中段景观塑造。完成建成区黑臭水体治理。提标改造9座污水处理厂，新建14处污水集中处理设施，完善重点区域污水管网，不断提高污水收集处理率、中水回用率。全面推行“河长制”。加快伊通河支流综合治理。完成“全国水生态文明城市建设试点”任务。基本完成三环以内旧城集中改造提升，坚持民生导向，突出改造危旧管网，完善老旧小区功能。优化提升历史街区，打造一批示范街路、精品商圈。新建续建6座公园，绿化提升一批主要街路，城区新增绿地100公顷。改造“暖房子”503万平方米。优化城区路网，完善“两横三纵”快速路，打通一批断头路、卡脖路、关键堵点。启动建设一批立体停车场。地铁1号线投入运营，地铁2号线实现热滑试车，加快建设轻轨北湖线、轻轨3号线延伸线。启动龙嘉机场综合交通枢纽建设。加快地铁6号线、机场快轨工程前期工作。创建“公交都市”示范城市，改革完善运营体制，调整优化线路，切实改善公共交通状况。启动新建六水厂前期工作。新增供热能力540万平方米，推动城市热网联接，提高居民供热质量。推进地下综合管廊建设。强化空气环境治理，基本淘汰“黄标车”，淘汰和治理超标排放燃煤锅炉，基本完成建成区重污染企业关停搬迁，秸秆综合利用率提高10个百分点。建成区机械化清扫率达到80%左右。实施城市管理综合执法改革，积极应用现代信息技术，不断提升城市管理水平。

　　（七）全力改善民生状况。继续实施幸福长春行动计划，努力办一批群众最关心、最直接、最迫切的民生实事。加大脱贫攻坚力度，确保贫困村、贫困人口全部脱贫，提前完成脱贫攻坚任务。积极扶持高校毕业生、农村转移劳动力、城乡就业困难人员和退役军人就业，加大创业担保贷款投放力度，城镇新增就业11万人。滚动实施“暖流计划”，促进城乡居民持续稳定增收。强化社会保险扩面征缴，稳步实施机关事业单位养老保险制度并轨。完善政府为空巢、失能、失独老人购买居家养老服务政策。大力发展民办养老机构，提高养老服务质量，新增养老床位3000张以上。移址新建市社会福利院。建立残疾、生活困难家庭学生生活补助制度。加大贫困精神病患者和农村贫困患者医疗救助力度。继续开展“万户特困户结对救助”等活动，及时救助群众各类突发性、紧迫性、临时性基本生活困难，坚决避免冲击社会道德和心理底线事件发生。加强农村留守儿童关爱保护。大力发展慈善、残疾人、志愿服务等公益事业。积极开展住房购租并举试点，努力让群众住有所居。新建8所义务教育学校，持续推动大学区改革、“强师计划”、“温馨村小”建设，不断提高义务教育质量。探索建立城区公办小学课后免费托管服务制度。整合居民医保与新农合制度，加快构建城乡统一的医疗保障体系。提高城镇职工医保报销限额、大额医疗救助标准。启动中省直医院综合改革，实现二级以上医疗机构检验结果互认。完善城区“10分钟体育健身圈”，倡导全民健身新时尚。繁荣文化市场，加强文化市场管理。深化群众性精神文明创建活动，不断提高市民素质和社会文明程度。

　　（八）强力维护安全稳定。始终牢记“生命至上、安全第一”，用最严格的监管、最严厉的处罚、最严肃的问责，推动城市安全水平不断提升。推进安全生产领域改革发展，完善安全生产防控体系，加强安全基础设施建设，强化风险分级管控与隐患排查治理，努力创建“国家安全发展示范城市”。深入推进“平安长春”建设，完善立体化、信息化社会治安防控体系，依法惩治违法犯罪行为，严密防范和严厉打击暴力恐怖活动，增强人民群众安全感。切实强化交通、消防安全管理，努力降低事故发生率和伤亡人数。深入创建“国家食品安全示范城市”。强化食品药品安全监管，源头严防、过程严管、风险严控，努力让人民群众饮食用药安全放心。建设社会综合治理信息平台。增强城市综合应急能力。充分利用信访机构接待、局长接待日、市长公开电话等渠道，调处纠纷、化解矛盾、促进和谐。完善落实重大信访案件包保制度，切实解决信访群众合理合法诉求。深入开展“七五”普法。推进法律服务惠民行动。进一步做好国家安全和民族宗教工作。支持深化国防和军队改革，深入开展双拥共建活动，推动军民融合深度发展。

　　（九）加强政府自身建设。深入贯彻落实新发展理念，把全面建成小康社会使命扛在肩上，把万家忧乐放在心头，努力建设群众满意的法治政府、创新政府、廉洁政府和服务型政府。依法接受人大及其常委会的监督，自觉接受人民政协的民主监督，接受社会和舆论监督，让权力在阳光下运行。坚持依宪施政、依法行政，所有行政行为都要于法有据。深入推进政务公开，及时回应社会关切，使群众了解政府做什么、怎么做。认真落实中央八项规定精神，坚持不懈纠正“四风”，深入开展反腐败斗争。加强行政监察，推进审计全覆盖。以减权限权、创新监管等举措减少寻租空间，铲除滋生腐败土壤。巩固拓展“三严三实”专题教育、“两学一做”学习教育成果，增强政治意识、大局意识、核心意识、看齐意识，加强作风和能力建设，打造高素质专业化的公务员队伍。健全并严格执行工作责任制，确保各项政策和任务不折不扣落到实处。强化督查问责机制，坚决整肃庸政懒政怠政行为。不断健全正向激励机制和容错纠错机制，给改革创新者撑腰鼓劲，让广大干部想干事、敢干事、能干成事。

　　各位代表！奋斗才能赢得未来。新的五年征程已经开始。让我们紧密团结在以习近平同志为核心的党中央周围，在省委、省政府和市委的坚强领导下，凝心聚力、锐意进取、扎实工作，为建设东北亚区域性中心城市而努力奋斗！
